# Structure of the dopamine D3 receptor bound to a bitopic agonist reveals a new specificity site in an expanded allosteric pocket

**DOI:** 10.21203/rs.3.rs-3433207/v1

**Published:** 2023-12-19

**Authors:** Sandra Arroyo-Urea, Antonina L. Nazarova, Ángela Carrión-Antolí, Alessandro Bonifazi, Francisco O. Battiti, Jordy Homing Lam, Amy Hauck Newman, Vsevolod Katritch, Javier García-Nafría

**Affiliations:** 1 Institute for Biocomputation and Physics of Complex Systems (BIFI) and Laboratorio de Microscopías Avanzadas (LMA), University of Zaragoza, 50018, Zaragoza, Spain; 2 Department of Quantitative and Computational Biology, University of Southern California, Los Angeles, CA 90089.; 3 Center for New Technologies in Drug Discovery and Development, Bridge Institute, Michelson Center for Convergent Biosciences, University of Southern California, Los Angeles, CA, USA.; 4 Medicinal Chemistry Section, Molecular Targets and Medications Discovery Branch, National Institute on Drug Abuse – Intramural Research Program, National Institutes of Health, 333 Cassell Drive, Baltimore, Maryland 21224, United States; 5 Department of Chemistry, University of Southern California, Los Angeles, CA, USA.

**Keywords:** G protein-coupled receptors, GPCR, bitopic drugs, cryo-EM, drug selectivity, dopamine D_3_ receptor

## Abstract

Although aminergic GPCRs are the target for ~25% of approved drugs, developing subtype selective drugs is a major challenge due to the high sequence conservation at their orthosteric binding site. Bitopic ligands are covalently joined orthosteric and allosteric pharmacophores with the potential to boost receptor selectivity, driven by the binding of the secondary pharmacophore to non-conserved regions of the receptor. Although bitopic ligands have great potential to improve current medications by reducing off-target side effects, the lack of structural information on their binding mode impedes rational design. Here we determine the cryo-EM structure of the hD_3_R coupled to a G_O_ heterotrimer and bound to the D_3_R selective bitopic agonist FOB02–04A. Structural, functional and computational analyses provide new insights into its binding mode and point to a new TM2-ECL1-TM1 region, which requires the N-terminal ordering of TM1, as a major determinant of subtype selectivity in aminergic GPCRs. This region is underexploited in drug development, expands the established secondary binding pocket in aminergic GPCRs and could potentially be used to design novel and subtype selective drugs.

## Introduction

While G protein-coupled receptors (GPCRs) form the largest family of drug targets, accounting for more than a third of FDA-approved drugs^1^, developing subtype-selective drugs is a major challenge. This is especially true for aminergic GPCRs, which include 42 receptors (dopamine, serotonin, adrenaline, histamine, acetylcholine and trace amine receptors) with high sequence similarity. In the most closely related aminergic receptor subtypes, sequence identity often exceeds 80% of the orthosteric binding site (OBS) residues. Such conservation supports neurotransmitter promiscuity^2^ between subtypes, but results in undesired off-target side effects of drugs that only bind in the OBS^3^. Controlling drug selectivity for aminergic receptors has the potential to improve current therapies and it could be achieved by the design of bitopic molecules^4–6^. These are ligands generated by covalently joining two pharmacophores, a primary pharmacophore (PP), that usually targets the OBS, and a secondary pharmacophore (SP), that targets an allosteric or secondary binding pocket (SBP) generally divergent in sequence and/or structure within the target receptor^4–7^. Hence, bitopic molecules have been proposed to have a separate “message-address” system wherein an agonist/antagonist, the *message*, is linked to a pharmacophore binding to the SBP, which contains the *address*^5,8^. Indeed, several bitopic compounds with enhanced receptor selectivity have been developed for GPCRs, specially for the muscarinic receptor where a variety of allosteric modulators is available^9–11^. Overall, bitopic ligands present a rational approach to develop molecules with enhanced functionality and selectivity, however there is scarce structural information on their binding modes and development relies heavily on classical structure-activity relationships^12^. Here we aim to understand the molecular basis of a selective bitopic drug that distinguishes between two closely related aminergic GPCRs, the human dopamine D_2_ receptor (D_2_R) and dopamine D_3_ receptor (D_3_R). These receptors share 78% sequence identity at the transmembrane segment and 100% identity at the OBS, making their pharmacological distinction a notoriously hard challenge^13,14^. D_2_R and D_3_R differ in brain distribution and signaling properties, and are both targeted by current antipsychotics and drugs for the treatment of neurological diseases (such as Parkinson’s disease^15,16^). Although agonists with some selectivity exist, treatments are still suboptimal due to a lack of selectivity and different effects originating from the activation of both receptors^17,18^. Hence, new subtype selective molecules are likely to help understand their physiological role as well as providing leads for improved therapeutics. Furthermore, selective activation of D_3_R may yield neuroprotective effects in Parkinsońs disease, hence harboring potential in the treatment of neurodegeneration^19,20^.

In this work, we determined the cryo-electron microscopy (cryo-EM) structure of the human D_3_R bound to a bitopic and full agonist (FOB02–04A) and coupled to a Gα_O_βγ heterotrimer. Together with functional assays, mutagenesis, docking studies, and molecular dynamics simulations, we determine the binding mode and basis for D_3_R selectivity of this compound. The bitopic molecule occupies the OBS and protrudes towards the outside of the ligand binding pocket to contact a new allosteric site at the extracellular vestibule of D_3_R formed by TM2-ECL1 and TM1. This region is of high sequence and structural variability and expands the established aminergic SBP, opening new avenues to develop subtype-selective bitopic drugs, potentially across other aminergic GPCRs.

## Results

### Overall cryo-EM structure of the hD_3_R:Gα_O_βγ:scFv16 bound to a bitopic ligand

**Figure 1.**
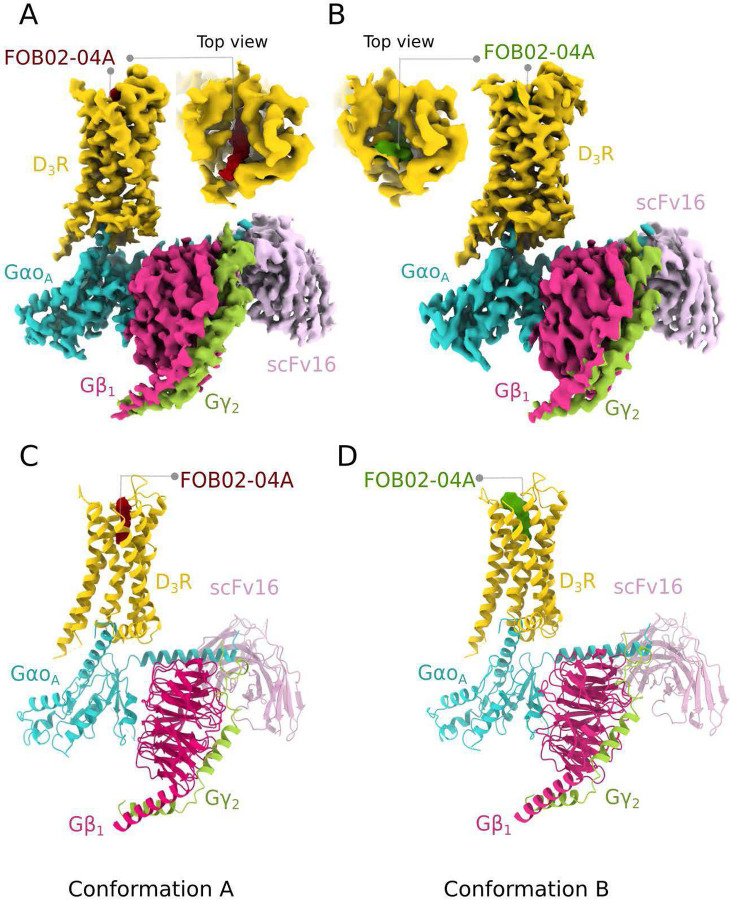
Overall cryo-EM reconstruction of the D_3_R-G_O_:FOB02–04A complex. Cryo-EM maps for the D_3_R-G_O_:FC)B02–04A complex in Conformation A **(A)** and B **(B)** are shown with an inset into the ligand binding site from the top view. Cryo-EM density is colored according to subunit with the bitopic ligand colored in red (Conformation A) and green (Conformation B). Coordinates for Conformation A **(C)** and B **(D)** for both complexes are shown as cartoons and colored by subunit with the bitopic ligand colored in red (Conformation A) and green (Conformation B).

The D_3_R:G_O_ heterotrimer:FOB02–04A complex was produced by co-expressing the hD_3_R (L^3.41^W mutation following Ballesteros–Weinstein numbering^21^), the dominant negative Gα_O_ subunit^22^, Gβ_1_ and Gγ_2_ in insect cells (see Methods) (Supplementary Fig. 1A). The D_3_R^L3.41W^ was previously used in structural studies^23^ and was validated in this work using cellular BRET assays^24^, where it displayed a virtually identical ligand-induced activation as the wild-type D_3_R (Supplementary Fig. 1B). The bitopic FOB02–04A was synthesized as previously described^9^ and was added before complex solubilization from insect cell membranes. The scFv16^25^ (which binds to the Gα_O_:Gβ interface) was incorporated prior to size exclusion chromatography. The structure of the complex was then solved by single-particle cryo-EM (Fig. 1 and Supplementary Fig. 2). Positioning the ligand binding pocket at the center of the cryo-EM box improved the resolution at the D_3_R extracellular region (Methods and Supplementary Fig. 3), allowing to separate two FOB02–04A and binding site conformations – Conformation A (to a global resolution of 3.05 Å) and B (global resolution of 3.09 Å), which mainly differed in the position of the bitopic SP and residues around the SBP (Fig. 1 and Supplementary Fig. 2). We will initially focus on Conformation A unless otherwise stated since Conformation B was concluded to be a non-productive antagonistic conformation (see below). Both final cryo-EM maps were of sufficient quality to build confidently the D_3_R, the Gαβγ proteins, the scFv16 and the bitopic FOB02–04A ligand (Supplementary Fig. 4 and Supplementary Table 1). Both D_3_R conformations were built from residues H29 (Conformation A)/Y32(Conformation B) to C400 with missing residues for intracellular loop 3 (ICL3) (residues I224 to R322, both excluded from the model). No cholesterol (or cholesterol hemisuccinate) or lipid molecules were found around the transmembrane part of the receptor, consistent with previous reported structures of the D_2_R^26–28^ and D_3_R^23,28,29^ and in contrast to D_1_R, D_4_R and D_5_R where cholesterol was bound to the transmembrane segment^28,30,31^.

### Activation mechanism and G_O_ coupling of the D_3_R bound to FOB02–04A

**Figure 2.**
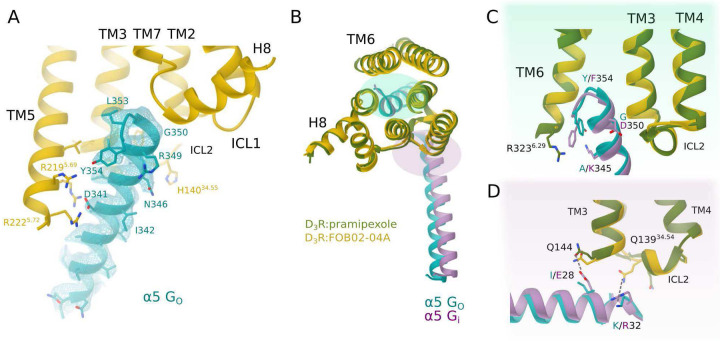
Coupling of the D_3_R to a G_O_ heterotrimer. Zooms into the D_3_R (yellow) interface with the C-terminal α5 of G_O_ (turquoise) or G_i_ (purple) shown as cartoons with relevant residues as sticks. **(A)** Interaction details of the D_3_R:G_O_ interface when bound to FOB02–04A. The cryo-EM density of the C-terminal α5 of G_O_ is shown as mesh. **(B)** Comparison of the D_3_R coupling interface to G_i_ (PDB 7CMU, green) and G_O_ (D_3_R-G_O_:FC)B02–04A). **(C)** C-terminal α5 interactions of G_O_ vs G_i_ (PDB 7CMU) coupling. **(D)** Interactions between ICL2 and intracellular section of TM4 with the αN of the G_i_ (PDB 7CMU, green) or G_O_ protein.

The D_3_R:Gα_O_βγ:FOB02–04A displays the characteristic structure of a GPCR:G protein complex, with resemblance to the previously determined structures of D_3_R coupled to a G_i_ heterotrimer^28,29^ (e.g. RMSD of 1.036 Å for 1022 Cα for the pramipexole bound structure, PDB 7CMU). No major conformational changes are found in the D_3_R when comparing it bound to pramipexole (PDB 7CMU), PD128907 (PDB 7CMV), rotigotine (PDB 8IRT) or FOB02–04A (0.535 Å RMSD over 253 Cα in the pramipexole bound as example) aside from the ordering of the extracellular region of TM1 (see below). The D_3_R activation induced by FOB02–04A follows the canonical conformational changes^32^, i.e. a downward shift of the toggle switch W342^6.48^, a conformational change of the PIF (I118^3.40^, F338^6.44^), DRY (D127^3.49^, R128^3.50^, Y129^3.51^) and NPxxY (N379^7.49^, P380^7.50^, P Y383^7.53^) motifs, which end up with an ~9 Å outward swing of the cytoplasmic end of TM6 and inward movement of TM7 towards the core of the receptor as compared to the inactive state^23^ (Supplementary Fig. 5). The coupling of the G_O_ heterotrimer to the D_3_R occurs through two interfaces: a first major interface located between the Gα_O_ C-terminal α5, that engages mainly with the intracellular part of TM3, TM5 and TM6 of the D_3_R (I344, L348, C351, L353, and Y354 in Ga_O_ packing against R128^3.50^, A131^3.53^, V132^3.54^, I2115.61, L2155.65, R2185.68, R2225.72, R3236.29, K3266.32, A3276.33 and M3306.36 in D3R) with contributions from TM7 and TM2 (Fig. 2A). Of note, from molecular dynamics (MD) simulations spanning five independent 0.6 μs runs of the D_3_R bound to FOB0204A and coupled to Gα_O_βγ within a membrane bilayer, alternating salt bridge interactions occurred between D341^G.H5.13^ (superscript denotes CGN numbering system^33^) of the Gα_O_ C-terminal α5 and the guanidinium groups of R218^5.68^ and R222^5.72^ in D_3_R (Supplementary Fig. 6). A second interface is located at the intracellular loop 2 (ICL2), which makes interactions in a pocket formed by the Gα_O_ N-terminal helix, the C-terminal α5 and the loops connecting the β-strands. The interaction is also held together by unspecific electrostatic charges between the receptor and the Gα protein conserved among G_i/O_ coupled receptors^28,29^.

The D_3_R has been shown to couple preferentially to G_O_ compared to G_i_^34^. The current D_3_R:G_O_ structure allows us to compare it with the previously determined D_3_R:G_i_ complex to search for potential differences that could explain such D_3_R coupling preference (Fig. 2B–C). Overall, both structures exhibit a similar interface area, with D_3_R-G_O_ having only a slightly lower buried surface area than D_3_R:G_i_ (959.4 Å^2^ and 1051.8 Å^2^ for G_O_ and G_i_ coupled D_3_R respectively). However, a smaller interface area is usually seen in G_O_ vs G_i_ couplings irrespective of selectivity^35,36^. Additionally, both structures present a similar outward swing in TM6 irrespective of G_O_ or G_i_ coupling (Fig. 2B–C), in line with previous observations of the same receptor coupled to different Gα proteins keeping the magnitude of TM6 outward swing^37,38^. However, differences occur when looking at the C-terminal a5 interactions of G_O_ *vs* G_i_. In the case of Ga_O_, the terminal Y354^G.H5.26^ points towards TM5, in contrast to its equivalent F354^G.H5.26^ in Gα_i_, which is sandwiched between R323^6.29^ and K345^G.H5.17^ (this residue is specific of Gα_i_, A345^G.H5.17^ in Ga_O_). As a result, D_3_R TM6 is shorter in the G_O_ coupled structure (Fig. 2C). Furthermore, previous studies suggested that native ICL contacts are essential to achieve G_O_ selectivity in D_3_R^34,39^. Indeed, structural differences were also found at the interaction made by ICL2 where, in G_O_, Q139^34.54^ moves away from the α5 of G_O_ to interact with K32 in the αN. Such interaction was further confirmed in MD simulations, whose interacting distance remained constant along the five trajectories spanning 0.6 μs each (Supplementary Fig. 6). Additionally, the interaction between Q144^ICL2^ and E28 in the G_i_ αN is lost when coupled to G_O_ due to a replacement of E28^G.HN.52^ by an isoleucine as well as the slight difference in the positioning of G_O_ with respect to the receptor (Fig. 2D). Overall, these differences might contribute to the D_3_R G_O_ selectivity.

### The binding mode of the bitopic agonist FOB02–04A at D3R

**Figure 3.**
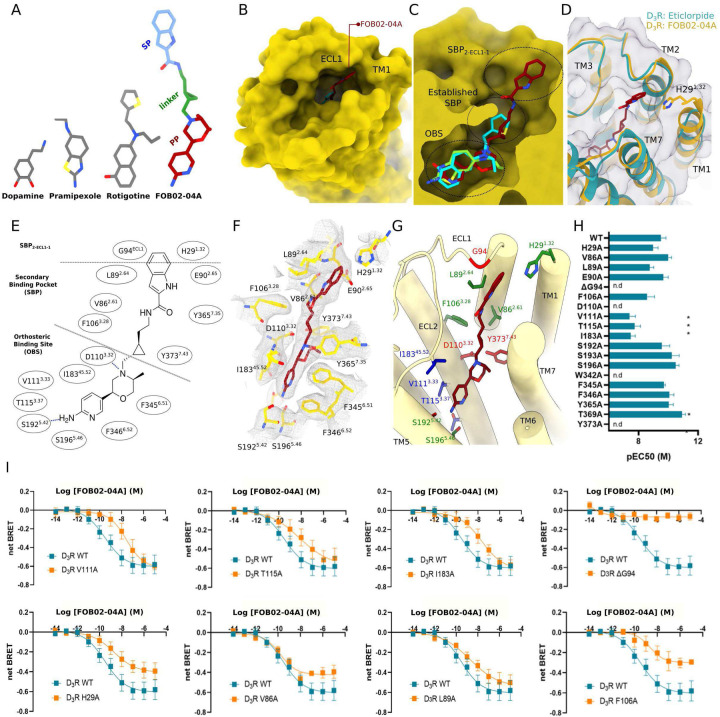
Binding of the bitopic FOB02–04A to the D_3_R receptor. **(A)** Schematic of dopamine, pramipexole, rotigotine and the bitopic FOB02–04A ligand shown as sticks and colored by component. **(B)** Binding of the secondary pharmacophore (SP) (sticks, dark red) to a groove-shaped pocket at the D_3_R (yellow, surface representation) formed by ECL1 and TM1. **(C)** Two views of a comparison of FOB02–04A (dark red carbon, sticks), pramipexole (green carbon, sticks) and rotigotine (cyan carbon, sticks) binding into the D_3_R pocket (yellow, surface representation). Dashed circles indicate OBS, established SBP and the new SBP_2-ECL1–1_ site. **(D)** Overall binding mode of the bitopic molecule to the D_3_R and ordering of TM1 upon bitopic binding. FOB02–04A (dark red, sticks) is displayed on superposed structures of D3R bound to eticlopride (cartoon, cyan) and FOB02–04A (cartoon, yellow) **(E)** Schematic of the FOB02–04A binding into the D_3_R ligand binding pocket. Residues at the OBS, SBP and SBP_2-ECL1–1_ are indicated. **(F)** Binding details of FOB02–04A (dark red, sticks) at the D_3_R (yellow sticks) with cryo-EM density as grey mesh. **(G)** Binding details of FOB02–04A (dark red, sticks) at the D_3_R (yellow cartoons) with residues at the ligand binding pocket colored by functional effect when mutated to alanine: decreased efficacy – green carbons, decreased potency – blue carbon and non-detectable binding – red carbon. **(H)** pEC50 values for alanine mutation of the residues at the ligand binding site in response to activation by FOB02–04A using the TRUPATH assay. All data are means ± SEM of three independent experiments performed in technical triplicate. *P< 0.05 (one-way ANOVA with Dunnett post hoc analysis), nd, non-detectable. (**I**) Dose response curves of D_3_R WT (blue curves) compared to dose response curves of relevant alanine mutants (orange curves) upon activation by FOB02–04A (shown as net BRET). Data are presented as means ± SEM of three independent experiments performed in technical triplicate.

Bitopic molecules are composed of a PP (binding at the OBS), an SP (binding at the allosteric site), and a linker. FOB02–04A is a full agonist bitopic molecule composed of a non-catechol PP (based on PF592,379, an aryl-morpholine-based scaffold), a SP with an indole-amide group, and a (1*R*,2*S*)-cyclopropyl linker moiety whose chirality has been optimized for ligand binding and selectivity^9,40,41^ (Fig. 3A). The cryo-EM density allowed modelling of the three bitopic components. Unlike other agonists, which target exclusively the bottom of the pocket, FOB02–04A binds to the OBS and runs along a narrow channel towards the allosteric site in the extracellular vestibule, interacting with residues from TM1–3 and TM5–7 (Fig. 3B–D). The SP of FOB02–04A is found protruding out of the tight channel to bind in the extracellular vestibule of D_3_R, occupying most of the ligand binding pocket, in contrast to pramipexole which only occupies 23% of the pocket volume (Fig. 3B–C). Each component of the bitopic molecule (PP, linker and SP) occupies a different region within the D_3_R pocket, overall defined by a combination of hydrophobic and polar interactions, as described in Fig. 3E.

The PP pocket at the OBS is defined by strong salt bridge interactions with D110^3.32^, and a cavity formed by S196^5.46^, F345^6.51^, F346^6.52^, W342^6.48^, V111^3.33^, T115^3.37^ and I183^ECL2^, with an additional weak H-bond with S192^5.42^ (Fig. 3). To correlate structural information with functional activity, most of the residues involved in ligand binding were mutated to alanine, following quantification of their surface expression and measurement of their ligand-induced activation using functional BRET assays in HEK293T cells^24^ (see Methods and Supplementary Fig. 7). At the OBS there were critical residues which showed no detectable activity when mutated to alanine such as the conserved D110^3.32^, which forms a stable charge interaction with almost all agonists in aminergic receptors, and W342^6.48^, the conserved toggle switch residue at the bottom of the OBS pocket that is essential for signaling. Additionally, I183^ECL2^, which sandwiches the ligand from the extracellular side (ECL2), V111^3.33^ and T115^3.37^ had a significant impact on agonist potency when mutated (Fig. 3H–I). V111^3.33^ is specifically relevant for FOB02–04A, since its mutation does not have an impact on the D_3_R-induced activation by pramipexole, rotigotine and PD128907^28,29^. In turn, T115^3.37^ is relevant for FOB02–04A and pramipexole in contrast to PD128907 and rotigotine. Finally, an agonist interaction with S192^5.42^ is found within most aminergic receptor-agonists pairs, however it seems to be less important for FOB02–04A binding (Fig. 3, Supplementary Fig. 7). This is in line with non-catechol agonists not relying heavily on S192^5.42^ for binding and activation^42^ (also observed for pramipexole^29^). A conserved hydrophobic pocket between T369^7.39^ and F345^6.51^ is efficiently occupied by the rotigotine, pramipexole and PD128907 propylamine group, while it is barely occupied by a methyl group by FOB02–04A (Supplementary Fig. 8). This may explain the lack of effect of F345^6.51^A upon activation by FOB02–04A and suggests that a larger hydrophobic group at this position might improve its binding.

The linker component of the FOB02–04A, which connects PP and SP, interacts with residues at the established SBP in aminergic receptors^3,12^, an unexploited region in pramipexole and PD128907 but occupied by the propylthiophene group in rotigotine^28^. The pocket is formed by residues V86^2.61^, F106^3.28^, T369^7.39^ and Y373^7.43^ and has been proposed to have different plasticity among dopamine receptors, and hence a source for ligand specificity^28^. In the case of FOB02–04A, three residues showed a significant reduction in activity when mutated to alanine: Y373^7.43^, F106^3.28^ and V86^2.61^. Y373^7.43^A showed non-detectable activity and, although this residue is known to be relevant for maintaining the D110^3.32^ geometry to make the conserved charged interactions with agonists, its mutation does not have such a pronounced effect on the activity of pramipexole, dopamine and PD128907^29^ as it has on the activity of rotigotine or FOB02–04A. This suggests a direct role in ligand-induced activation of the bitopic molecule. Additionally, alanine mutation of F106^3.28^ and V86^2.61^ showed reduced efficacy. This is likely to be FOB02–04A specific since V86^2.61^A did not reduce efficacy upon pramipexole activation^29^. Overall, the linker connecting the PP and SP has an active role in the D_3_R selective binding and function of FOB02–04A and its related bitopic analogs^29^.

Finally, the FOB02–04A SP binds in a groove-shaped pocket at the receptor extracellular region, denoted as SBP_2-ECL1–1_ and formed by the tips of TM1 and TM2, and ECL1. Remarkably, in contrast to prior D_3_R structures—whether in active or inactive conformations—the outermost extracellular residues undergo a rearrangement that positions H29^1.32^’s imidazole group, situated between TM2 and TM7, to stack with the 1H-indole group of the ligand SP. Given the absence of H29^1.32^ in preceding D_3_R cryo-EM^28,29^ and crystal structures^23^, we sought to ascertain the orientation of the imidazole moiety of H29^1.32^. For this purpose we performed comparative MD simulations, involving two D_3_R complexes coupled to Gα_O_βγ and bound to either FOB02–04A or pramipexole (PDB ID: 7CMU), both within a membrane bilayer and aqueous milieu and executed across five parallel runs of 0.6 μs each. MD analysis elucidated a more consistent localization of H29^1.32^ between TM2 and TM7 when complexed with FOB02–04A relative to pramipexole. In this conformation, H29^1.32^ side chain is directed towards the SBP_2-ECL1–1_, engaging with the SP of FOB02–04A bitopic ligand (Supplementary Fig. 9). Although the protonated N(ε) atom of H29^1.32^ imidazole and the carboxyl entity of E90^2.65^ are too distant to support strong polar or ionic interactions, the D_3_R complex with the bitopic ligand FOB02–04A exhibited a narrower distance distribution than in pramipexole complex (Supplementary Fig. 9). In addition, in the D_3_R-FOB02–04A complex, the N(ɛ) atom of H29^1.32^ consistently interacts with the backbone oxygen of E90^2.65^. Conversely, when complexed with pramipexole, three of the five trajectories show this distance consistently surpassing 10Å. This observation reinforces that, while in the FOB02–04A:D_3_R complex the H29^1.32^ side chain is predominantly positioned in the SBP_2-ECL1–1_ where it is stabilized by the ligand, in the pramipexole-bound complex H29^1.32^ side chain points away, likely due to the absence of the allosteric pharmacophore in pramipexole (Supplementary Fig. 9).

To gain further insights into the SBP_2-ECL1–1_ role, we mutated all residues within this pocket to alanine (except for G94^ECL1^ which was deleted) and measured ligand-induced activation using BRET2 assays. These experiments revealed that deletion of G94^ECL1^, which prevents ECL1 from reaching the SP is essential for FOB02–04A activity (Fig. 3H–I). A previous study identified G94^ECL1^ as a key determinant for binding of a similar bitopic molecule, however, only a reduction in affinity was observed (using radioactive ligands and fluorescence)^43^ while, in the current study, ligand-induced activity seemed to be fully ablated. This suggests that FOB02–04A could potentially still bind in the DG94^ECL1^ variant (although with lower affinity) but triggers no detectable Gαβγ activation, hence G94^ECL1^ is likely to determine affinity and efficacy. Further mutational analysis of residues within SBP_2-ECL1–1_ identified L89^2.64^ and H29^1.32^ as key residues, with only a slight reduction in potency (~3-fold and ~6-fold reduction in EC_50_ for H29^1.32^ and L89^2.64^ respectively) but a significant decrease in efficacy upon alanine mutation (especially for H29^1.32^) (Fig. 3I). Previous studies predicted how slight variations in the position of the PP at the D_3_R OBS could modulate compound efficacy^44^. Since several residues at the SBP modulate FOB02–04A efficacy, it is likely that the linker and SP conformation are currently optimal to position the PP for maximal efficacy at the OBS, and that mutations around the SBP restrict conformations of the FOB02–04A PP to less efficacious alternatives. This scheme yields a marked segregation of the functional roles of the protein residues for each bitopic component. While mutations significantly decreasing potency (>100-fold the EC_50_) are primarily found at the OBS, mutations at the SBP mainly decrease FOB02–04A efficacy (Fig. 3G). This suggests that the SP is not only involved in D_3_R selectivity (see below) but also in optimally positioning the PP for activity. Such conclusions are in line with previous suggestions originating in computational and functional assays^45^. Interestingly, additional controls where activation of the H29^1.32^A variant was tested with pramipexole, an agonist that does not reach H29^1.32^, also displayed a reduction in efficacy (data not shown). Hence, we could not quantify the contribution of this residue on bitopic binding since it readily displayed an effect on the intrinsic receptor efficacy. It is not unusual for residues at the most extracellular sites to have an impact in intrinsic receptor function^46^.

Additional analysis of the MD trajectories with the D_3_R-FOB02–04A complex suggested a more robust interaction of FOB02–04A with D_3_R than pramipexole. This was observed by looking at the stable salt bridge interaction between the trans-cyclopropyl amine group of FOB02–04A and the carboxyl group of D110^3.32^ in D_3_R (which underscores the stable binding pose of the 6-(aminopyridin-3-yl)-5-methylmorpholine PP moiety) (Supplementary Fig. 6). However, for the pramipexole-bound D_3_R complex, three out of five MD trajectories displayed substantial deviations in either the equivalent salt bridge interaction with pramipexole amino group, as well as the interactions distance between S196^5.46^ in D_3_R and the pramipexole’s amino group. Since pramipexole and FOB02–04A have similar binding affinities, the propensity of pramipexole towards dissociation observed during MD simulations suggests potential faster association and dissociation rates, in line with the larger bitopic molecule requiring longer times for association and dissociation (Supplementary Fig. 6).

Overall, the bitopic agonist FOB02–04A uses all three components (PP, linker and SP) to make critical interactions with the ligand binding pocket, since each component contributes with one critical interaction which, if mutated, the ligand-induced activation as a whole, is eliminated. This highlights that selective bitopic molecules are required to bind *en bloc* and that the SP which contains the *address* component is required to contribute significantly to the overall ligand function, otherwise selectivity would be lost.

### Structural basis of FOB02–04A D_3_R/D_2_R selectivity

**Figure 4.**
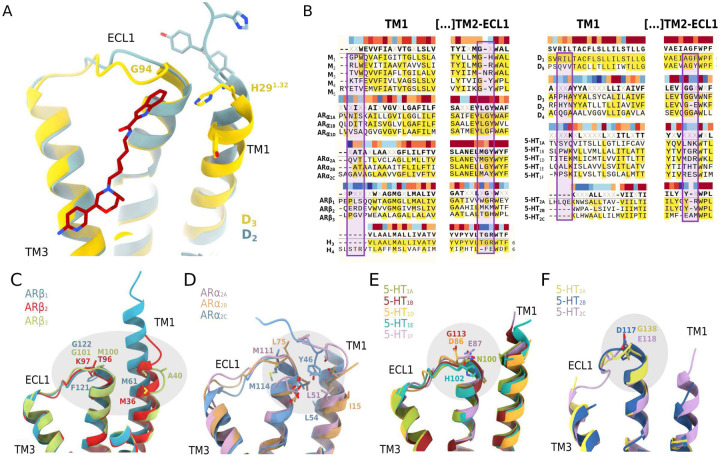
Sequence and structural diversity of the SBP_2-ECL1–1_ in aminergic GPCRs. **(A)** Comparison of the D_3_R (yellow cartoons with relevant residues as sticks) and D_2_R (light blue cartoons with relevant residues as sticks) TM2-ECL1 and TM1 regions within reach of FOB02–04A (dark red, sticks), **(B)** Sequence alignment of TMl and TM2-ECL1 regions in aminergic GPCRs with residues around the SBP_2-ECL1–1_ embedded in a box. Sequence conservation is color-coded above each residue position (gradient from dark red, conserved, to dark blue, non-conserved). Structural differences at the SBP_2-ECL1–1_ site among closely related adrenergic receptors **(C, D)** and serotonin receptors **(E, F).** Receptor are shown as cartoons colored by receptor with relevant residues shown as sticks.

The bitopic FOB02–04A ligand has been designed for its PP to carry the agonist *message* while the SP carries the *address*, and has been reported to be 50-fold more selective for D_3_R over D_2_R^9^. Since quantification of selectivity at the D_3_R/D_2_R is assay and condition-dependent^6,9,13^, we measured the D_3_R/D_2_R selectivity using cellular BRET assays, which confirmed the 50-fold selectivity (Supplementary Fig. 7). D_3_R and D_2_R have 78% sequence similarity at the transmembrane region and, residues within interacting distance of FOB02–04A, showed high structural similarity and 100% sequence identity at the OBS and established SBP^24^. However, FOB02–04A interactions with the G94^ECL1^ and H29^1.32^ within the SBP_2-ECL1–1_ form a region that is structurally and sequence diverse between D_3_R/D_2_R. The D_3_R TM2-ECL1 harbors an extra glycine residue that is absent in D_2_R (93GGV95 in D_3_R vs 98GE99 in D_2_R), which allows this region to interact with the SP in the D_3_R and not in the D_2_R (Fig. 4A). Deletion of the extra glycine G94^ECL1^ in D_3_R ablates ligand induced activation by FOB02–04A (Fig. 3I and Supplementary Fig. 7). This is in line with previous studies where similar bitopic molecules showed reduced affinity in D_3_R lacking G94^ECL1 43^. This reduction in activity makes G94 the most critical residue for D_3_R/D_2_R selectivity. Additionally, H29^1.32^ is positioned in TM1, the most sequence diverse transmembrane helix in GPCRs and that, within D_3_R/D_2_R, shows both sequence and structural diversity (Fig. 4A). Further exploiting this unforeseen H29^1.32^ has the potential to contribute with selectivity at the functional level.

### Diversity of the SBP2-_ECL1–1_ in other aminergic receptors

There are 9 groups of (clinically relevant) closely-related aminergic receptors sub-types (M_1–5_, ARα_1A-1D_, ARα_2A-2C_, ARβ_1–3_, D_1_ and D_5_, D_2_-D_4_, H_3–4_, 5-HT_1A-1F_, 5-HT_2A-2C_) for which sequence similarity poses problems to generate subtype selective ligands. Selectivity can arise from sequence diversity, structural divergence as well as differences in structural plasticity. Using sequence alignments and the recent explosion in GPCR structural information we assessed whether the SBP_2-ECL1–1_ is a site of high diversity that could be exploited to develop subtype selective drugs in other aminergic GPCRs. The analysis showed that the SBP_2-ECL1–1_ is variable either in sequence, structure or both within most aminergic receptor subtypes (Fig. 4B–F). The amount of diversity at the SBP_2_-ECL1–1 varies within each subfamily, with the least variable being the muscarinic receptors where TM1 is too far apart to contribute in all available structures and the equivalent G94 position is only different in M_3_R (N131^ECL1^ vs a glycine residue in M_1_, M_2_, M_4_ and M_5_). However, there are marked differences in several other subgroups. First, the serotonin 5-HT_1_ and 5-HT_2_ groups show variable sequence or structure at the G94^ECL1^ equivalent position while TM1 is too far apart (Fig. 4C and D). Additionally, the recent structural determination of all five dopamine receptors (D_1_R-D_5_R) highlighted the SBP_2-ECL1–1_ as the most variable region between them^28^. Finally, there are groups with marked differences at the SBP_2-ECL1–1_ site, e.g. the ARα_2A-2C_ subgroup. ARα_2A-2C_ show differences at the G94^ECL1^ equivalent position, while they have an increasingly ordered TM1 which could potentially contribute with specific interaction in each receptor. While in ARα_2B_ TM1 is far apart, it is longer in ARα_2A_ where it could contribute with main chain atoms of Y43 and in ADa_2C_ where the N-terminus folds over the TM2-ECL1 site providing with additional specific residues (Fig. 4D). In ARβ_1–3_, the TM2-ECL1 has structural and sequence divergence that could be used to design highly subtype selective bitopic molecules (Fig. 4C). Overall, the SBP_2-ECL1–1_ site is a major specificity region that is underexploited for developing subtype selective drugs. However, this site is far away from the canonical ligand binding site and might be better accessible with bitopic molecules.

### Alternative FOB02–04A conformation at the ligand binding site

**Figure 5.**
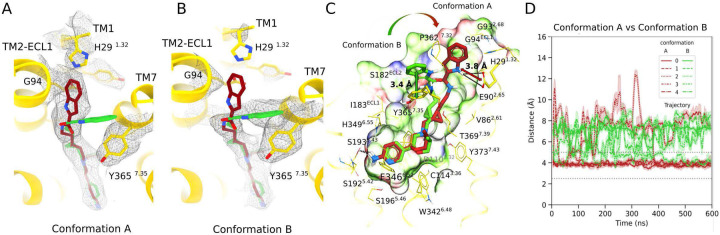
Conformation A and B within the D_3_R-G_O_:FC)B02–04A complex. Coordinates of the D_3_R (yellow cartoon, with relevant residues as sticks) are shown with FOB02–04A in Conformation A (dark red, sticks) superposed to Conformation B (green, sticks), Cryo-EM density is shown as grey mesh for Conformation A **(A)** and Conformation B **(B)** with both superposed FOB02–04A conformations. **(C)** Predicted binding poses of bitopic FOB02–04A with D_3_R showing Conformation A and Conformation B with intramolecular interactions shown as black dashes lines. Black arrows indicate distances for assessing bitopic FOB02–04A binding pose distribution between Conformations A and B with specified closest distances (E90^2.65^ carboxyl group in D_3_R to FOB02–04A indole atom N5 and from Y365^7.35^ 4-hydroxyphenyl moiety in D_3_R to the phenyl ring of FOB02–04A lFI-indole-2-carboxamide SP). A semi-transparent skin reveals the receptor molecular surface, which is colored by the residue properties (red (acidic), blue (basic), green (hydrophobic)). **(D)** Interaction dynamics between D_3_R E90^2.65^ and FOB02–04A SP (depicted in brown pallet) compared with proximity distance between D3R Y365^7,35^ and the SP of FOB02–04A (shown in green palette) suggest that FOB02–04A predominantly adopts Conformation A over B. Data from five independent simulations of D_3_R-Gαoβγ heterotrimer crystal complex are shown, spanning 0.6 μs of cumulative time per system, with the sampling rate of 10 frames per ns, solid lines and same-color shadows representing moving average values and one standard deviation respectively from 50 frames in all cases.

Docking of FOB02–04A to the D_3_R reliably reproduced its binding mode when compared to the cryo-EM structure. Yet, a second conformation of FOB02–04A was revealed with comparable docking scores, suggesting a second plausible orientation (Fig. 5C). In the alternative binding mode, termed Conformation B, the 1H-indole-2-carboxamide SP of FOB02–04A is seen to interact with a less hydrophobic pocket defined by the polar side chains S182^ECL2^, Y365^7.35^, as well as V360^ECL3^ and P362^7.32^ residues, termed hereafter SBP_ECL2-ECL3_. Notably, π-π stacking interactions between the 1H-indole part of FOB02–04A and Y365^7.35^ stabilizes Conformation B (Fig 5). MD simulations indicated that the indole SP of FOB02–04A oscillates between SBP_2-ECL1–1_ (Conformation A) and the comparatively less hydrophobic SBP_ECL2-ECL3_ (Conformation B). A detailed examination of the proximity between D_3_R E90^2.65^ and the FOB02–04A SP (accentuated with a red palette) juxtaposed with proximity measurements between D_3_R Y365^7.35^ and the FOB02–04A SP (illustrated in green pallet) provides insights into the temporal predominance of FOB02–04A’s Conformation A versus Conformation B (Fig 5D). Subsequent frequency analyses showed that Conformation A, that engages SBP_2-ECL1–1_, is predominant with an estimated 80% prevalence, in contrast to the 20% observed for Conformation B, targeting SBP_ECL2-ECL3_ region (Figure 5D and Supplementary Fig 6). This information triggered a targeted search for Conformation B within the cryo-EM dataset, which resulted in a model at 3.09 Å resolution (Fig. 1, Supplementary Fig. 2 and 4). In this model, cryo-EM density supports the second conformation for the FOB02–04A SP so as to make π-π stacking interactions with Y365^7.35^ in a similar manner as found in docking and MD simulations (Fig. 5A–B). Interestingly, in this cryo-EM map, the extracellular residues of TM1, including H29^1.32^, are not resolved, reminiscent of the pramipexole, rotigotine and PD128907 bound D_3_R structures (Fig. 5B). This suggests that binding of the SP to the SBP_2-ECL1–1_ stabilizes the TM1 conformation described above (in agreement with our MD simulations). A comparison of particle numbers between cryo-EM models of Conformation A and B also supported a predominance of Conformation A over B (~60%). With such relative abundance both conformations are expected to contribute to function, however, mutating Y365^7.35^ to alanine (affecting only Conformation B) did not have an impact while the DG94^ECL1^ variant (affecting only Conformation A) fully ablated Gαβγ dissociation. Such functional outcome does not respond to two functional conformations in equilibrium, but rather we propose that this second conformation is acting as an antagonist or weak partial agonist. Such hypothesis would be in line with our previous observation that residues at the SBP as well as the position of the SP are highly relevant for an optimal positioning and efficacy of the PP. In support of this, a minor twist of the PP at the OBS is observed in Conformation B with respect to Conformation A, and minor modifications at the position of the ligand at the D_3_R OBS have been shown to regulate ligand efficacy. However, we cannot rule out that the slight difference in PP position is a consequence of the low map resolution. If Conformation B was truly an antagonist, and these two conformations are in equilibrium, mutation of Y365^7.35^ should drive all the ligand into the first conformation A, and an increase in potency should result (since Conformation B acts like an antagonist). Indeed, a slight increase in potency was observed in the Y365^7.35^A (Fig. 3 and Supplementary Fig. 7), but it was not statistically significant. However, both conformations have similar relative abundance, therefore a major impact in activity is not expected. We then assessed whether Conformation B could be occurring at D_2_R, since this residue is conserved in D_2_R and could account for the binding affinity of FOB02–04A at D_2_R. However, Y408^7.35^A in D_2_R did not result in a signaling loss in functional assays (Supplementary Fig 7). Since D_2_R is more plastic than D_3_R, the binding mode of this bitopic molecule to D_2_R might be hard to predict and additional studies would be required. Overall, this second conformation of the bitopic molecule highlights that large flexible molecules might adopt alternative non-productive conformations that might hinder progress in drug development if non-detected.

## Discussion

Aminergic receptors are highly relevant drug targets, but the high sequence and structural similarity within the family poses a great challenge to developing subtype-selective drugs. Here, we have reported the cryo-EM structure of the human D_3_R in complex with the D_3_R-selective bitopic agonist, FOB02–04A, and coupled to a G_O_ heterotrimer. FOB02–04A binds D_3_R with all three components (PP, linker and SP), fully exploiting the OBS, established SBP and a new extended SBP_2-ECL1–1_ that confers FOB02–04A with D_3_R selectivity. This SBP_2-ECL1–1_ is structurally and/or sequence diverse also in aminergic receptors and could potentially be used to develop subtype-selective ligands. Especially interesting is the TM1 contribution to ligand binding since it is the most sequence diverse transmembrane region in GPCRs, rarely contributes to ligand binding, and could be exploited through the use of bitopic molecules with the required composition and length. Mutational profiling of the ligand binding site showed a marked segregation in functional roles of the residues at the OBS and the SBP. While the majority of mutations that impaired potency were located mainly at the OBS, mutations that impaired efficacy were enriched at the SBP. This highlights the relevant role of the SP binding in optimally positioning the PP at the OBS for maximal activity. The computational design of bitopic molecules might benefit from taking such roles into consideration. Additionally, the mutational analysis pointed to a mutually PP, linker and SP-dependent binding mode, i.e. all components contribute with essential interactions for the *en bloc* binding of the bitopic molecule. This is likely required when higher selectivity is desired since independent binding might yield promiscuous PP binding. Therefore, the *message* and *address* components should not be treated as separate entities when developing specific bitopic molecules, but rather working together in tandem with the appropriate linker in between^6^.

A second antagonistic conformation of the FOB02–04A bitopic molecule is proposed which suggests that care should be taken when developing subtype selective bitopic molecules, since the position of the PP at the OBS seems to be altered easily (at least for the D_3_R in the case of FOB02–04A) and bitopic molecules tend to be large and flexible, and alternative non-productive conformations might obscure highly specific and potent conformations in functional assays. Such problems likely contribute to the challenges associated with developing agonistic bitopic molecules^6^. Including structural determination in the drug development pipeline is likely to accelerate future progress. Additional structural information on other bitopic-receptor complexes might shed light on this topic.

Regarding the D_3_R/D_2_R selectivity, a recent report describing the structures of the five dopamine receptors (D_1_R-D_5_R) pointed towards H^6.55^ as a specificity determinant, since this residue changes conformation between D_2_-like receptors in an agonist-dependent manner^28^. While H^6.55^ was located far away from the bitopic molecule under study, molecules with combined interactions at H^6.55^ and the new extended SBP_2-ECL1–1_ site have the potential to yield highly specific molecules within D_2_-like receptors. Such molecules could help to improve current treatments targeting the D_3_R, a current target for Parkinsońs disease and other neurological disorders and neuropsychiatric disorders, including substance use disorders^47–49^.

Overall, this work extends the usable SBP in aminergic receptors exploiting an extracellular region of high sequence and structural variability and highlights new insights and pitfalls into the development of highly selective subtype selective bitopic molecules with desired functional efficacies.

## Methods

### Construct design and molecular cloning

All mutagenesis and molecular cloning procedures were performed using the *in vivo* DNA assembly method^350,51^. The cDNAs of the human D_3_R (HASS-FLAG-EGFP-3C_protease_-D_3_R with the L^3.41^W mutation) and human dominant-negative Gα_OA_ subunit (S47N, G204A, E246A, M249K and A326S)^22^ were obtained through gene synthesis (Gene Fragments, Twist Bioscience) and cloned into the pBacPak8. Rat His_8_-Gβ_1_ (pBacPak8), human Gγ_2_ (C68S) (pBacPak8) and a baculovirus expressing the scFv16 with a gp67 secretion signal and a C-terminal His_8_-tag were a gift from Christopher G. Tate’s laboratory. For BRET assays in HEK293T cells the same human HASS-FLAG-EGFP-3C_protease_-D_3_R construct was sub-cloned into the pcDNA4/TO vector, upon which all mutants were made (including the wild-type D_3_R). Constructs containing the Gα_OA_-RLuc8, Gβ_3_ and Gγ_9_-GFP2 in pCDNA5 and pCDN3.1 were a gift from Bryan Rothś lab (Addgene plasmid kit # 1000000163).

### D_3_R:Gαβγ:scFv16 production and purification

The scFv16 was produced by infecting *Trichoplusia ni* (Tni) cells grown in ESF921 media (Expression Systems) at a density of 2–3×10^6^ cells/ml and incubated for 48 h at 29 °C. The supernatant was clarified by centrifugation, dialysed to 20 mM Tris-base pH 8, 500 mM NaCl and 20 mM imidazole and loaded onto a pre-equilibrated HisTrap excel column (Cytiva). The scFv16 was eluted with an imidazole linear gradient, concentrated and loaded onto a Superdex 200 10/300 GL increase column (Cytiva) equilibrated in 20 mM HEPES pH 7.5 and 100 mM NaCl. Pure protein was concentrated to 4.2 mg/ml, flash-frozen and stored at −80 °C until further use.

For the production of the D_3_R:Gαβγ protein complex, recombinant baculoviruses expressing D_3_R, Gα_OA_, Gβ_1_ and Gγ_2_ were prepared using the FlashBAC ULTRA^®^ system (Oxford Expression Technologies). Tni cells were grown in suspension in ESF921 media to a density of 2–3×10^6^ cells/ml, co-infected with D_3_R, Gα_OA_, Gβ_1_ and Gγ_2_ baculoviruses (1:1:1:1 ratio) and shaked at 29 °C for 48 h. Cells were harvested, flash frozen in liquid nitrogen and stored at −80°C for further use. Cell pellets were thawed in 20 mM HEPES pH 7.5, 150 mM NaCl, 10% glycerol, 20 mU/mL apyrase and protease inhibitors cocktail (10 μM E-64, 0.05 μg/ml aprotinin, 0.02 μg/ml leupeptin, 10 μM benzamidine HCL, 0.01 μg/ml pepstatin, 10 μM bestatin and 10 μM PMSF) and incubated with 10 μM of FOB02–04A (compound 53a in ref.^9^) for 30 minutes at 4 °C. Cells were then solubilized with 0.5% (w/v) lauryl maltose neopentyl glycol (LMNG, Anatrace) supplemented with 0.071% (w/v) cholesterol hemisuccinate (CHS, MP Biomedicals ^™^) at 4 °C for 1 h. The sample was clarified by centrifugation and the supernatant was incubated with Talon Superflow (GE Healthcare Life Sciences) resin overnight at 4°C. The resin was then washed with 20 column volumes of 20 mM HEPES pH 7.5, 150 mM NaCl, 10% glycerol, 0.007:0.001% LMNG/CHS, 10 μM FOB02–04A and 5 mM imidazole followed by 20 CV of the same buffer with 20 mM imidazole. The sample was eluted with 20 mM HEPES pH 7.5, 150 mM NaCl, 10% glycerol, LMNG 0.003%, 10 μM FOB02–04A and 250 mM imidazole. The complex was concentrated and incubated with pure scFv16 at a molar ratio of 1:1.1 (D_3_R:Gαβγ_:_FOB02–04A to scFv16) at 4 °C for 30 minutes. The resulting complex was purified with a Superose 6 Increase 10/300 GL column (Cytiva) equilibrated with 20 mM HEPES pH 7.5, 25 mM NaCl, 0.003:0.0004% LMNG/CHS and 10 μM FOB02–04A. Pure protein was concentrated at 2.8 mg/ml and FOB02–04A ligand was added to a final concentration of 50 μM.

### Cryo-grid vitrification and data collection

3 μl of D_3_R:Gαβγ:FOB02–04A:scFv16 at 2.8 mg/ml were applied to 300 mesh Quantifoil 0.6/1 Au grids previously glow discharged with a Leica EM Ace200 Vacuum Coater at 15 mA for 60 s and vitrified with ethane using a Vitrobot Mark IV (FEI Company). Data collection was carried out in a Titan Krios at 300kV using a K3 detector at the European Synchroton Radiation Facility (ESRF). A total of 22,655 movies were recorded at a magnification yielding 0.84 Å/pixel with a dose rate of 17.6 e^−^/pixel/s and a defocus range between −1 to −3 μm using the Smart EPU Software (ThermoFisher Scientific). Movies were split into 50 frames each and exposed to a total dose of 50 e^−^/Å^2^ (1e^−^/Å^2^ per frame) using a total exposure time of 2 s.

### Cryo-EM Data processing

RELION-4.0^52^ was used for all data processing unless otherwise specified. Drift and beam-induced motion correction (5 × 6 patches) were performed using MotionCor2^53^ along with dose weighting. Contrast transfer function (CTF) estimation and determination of defocus range were performed with CTFFIND-4.1^54^. Automated particle picking was carried out with Topaz^55^. The initial particles were reduced to 475,951 after 2 rounds of 2D and 3D classifications (using an *ab initio* model). The best model was refined and subjected to CTF refinement and Bayesian polishing following a 3D classification focused on the receptor (with a mask around the receptor) that yielded 429,908 particles. Refinement of this set of particles yielded a model at 3.16 Å but poor cryo-EM density at the SBP. To improve map quality at the ligand binding site two parallel processing paths were pursued with the 429,908 particle set: 1) a recentering of the particles at the ligand binding site (re-extracted in a 320-pixel box) followed by 3D classification (resulting in 360,038 particles), and 2) 3D classifications with a mask at the extracellular half of the receptor followed by a recentering of the particles at the ligand binding site (as described before) which were further 3D classified (resulting in 176,315 particles). The two sets of particles were merged and duplicates removed, yielding 275,383 particles which were refined using for the last iteration a mask that precluded the Gα_O_-helical domain and the micelle. Post-processing resulted in a cryo-EM map for Conformation A at 3.05Å. Conformation B was obtained by performing a 3D classification on the 429,908 particle set with a mask on the extracellular half of the receptor resulting in a model with 252,959 particles which were subsequently re-centered at the ligand binding site and further 3D classified. Particles belonging to best model, with 201,219 particles, were subjected to heterogeneous refinement and 159,184 particles were lastly refined through non-uniform refinement in CryoSPARC^56^. This resulted in a cryo-EM map at 3.09 Å according to the gold-standard FSC of 0.143. Local resolution was calculated using CryoSPARC for both models.

### Model Building

Model building and refinement was carried out using the CCP-EM software^57^ and Phenix^58^. The D_3_R, Gβ_1_, Gγ_2_ and scFv16 starting coordinates were taken from the Gα_i_-coupled D_3_R structure (PDB code 7CMV)^29^. The Gα_O_ starting coordinates were taken from the Gα_O_-coupled α_2β_ adrenoreceptor structure (PDB code 6K41). D_3_R was modelled from residue H29 to I223 and from R323 to C400 in conformation A (conformation B starts at Y32). Gα_O_ was modelled from T4 to K54, T182 to V234 and N242 to Y354. Jelly-body refinement was performed in REFMAC5^59^ followed by manual modification and restraint real space refinement in Coot^60^ and Phenix. A dictionary describing the ligand FOB02–04A and its coordinates was created using AceDRG^61^ and manually fitted into the density for its latter refinement in real space using Coot and Phenix. B factors were reset to 40 Å^2^ prior to refinement. The final model achieved good geometry (Supplementary Table 1) with validation performed in Coot, EMRinger^62^ and Molprobity^63^. The goodness of fit of the model to the map was carried out using Phenix, using of a global model-*vs*-map FSC correlation (Supplementary Table 1).

#### Cellular BRET assays

pEC_50_ were determined using cellular BRET2 assays with the TRUPATH system^24^. 50,000 cells/well were seeded in previously poly-lysined 96-well white plates with clear bottom. The following day, cells were transfected with TransIT-2020 (Mirus Biosciences) at ratio of 2:1:1:1 of D_3_R:Gα_OA_-RLuc8:Gβ_3_:Gγ_9_-GFP2 (7:1:1:1 for D_3_R^Y373A^, D_3_R^ΔG94^ and D_2_R^Y408A^) following manufacturer instructions. After 48 h, the medium was replaced by 90 μl/well of freshly prepared assay substrate buffer (1×Hank’s balanced salt solution, 20 mM HEPES pH 7.4, Coelenterazine 400a 7.5 μM). 10μl of each concentration of FOB02–04A was added and the plate was read using a CLARIOstar (BMG Labtech) with 400 nm (RLuc8-Coelenterazine) and 498.5 nm (GFP2) emission filters at integration times of 1.85 s. BRET ratios were calculated as the ratio of GFP2 signal to Rluc8 signal. Data analysis was performed using GraphPad Prism 8.0.1. Data were normalized and a four-parameter logistic curve was fit into the data. Data are presented as mean ± SEM of three independent experiments performed in technical triplicate.

#### Surface expression quantification

HEK293T cells were plated in previously poly-lysined 96-well white plates (50,000 cells/well) and transfected the next day with the D_3_R and D_2_R variants using PEI MAX^®^ at a 2:1 ratio (PEI:DNA). After 48 h, cells were washed twice with 1X Phosphate Buffered Saline (PBS) and fixed with 4% paraformaldehyde for 20 minutes at RT. Cells were then washed three times with PBS for 5 minutes and 100 μl of 1X PBS with 5% BSA (w/v) was added to each well and incubated at RT for 30 minutes. Subsequently, media was replaced with 1X PBS-5% BSA with an anti-Flag HRP conjugate (1:10,000) and incubated at RT for 30 minutes. Cells were then rinsed twice with PBS and 50 μl of HRP substrate (Clarity Max^™^ Western ECL Substrate) was added to each well and incubated for 5 minutes prior to chemiluminescence detection using a CLARIOstar (BMG Labtech). Data analysis was performed using GraphPad Prism 8.0.1 Chemiluminescence values were normalized to D_3_R WT and presented as a ratio of D_3_R WT. Data are presented as mean ± SEM of three independent experiments performed in technical triplicate.

#### Molecular Dynamic simulations

The Gromacs simulation engine (version 2020.3)^64^ was used to run all molecular dynamics simulations under the Charmm36 force field topologies and parameters^65,66^. Charmm force field parameters and topologies for the ligands FOB02–04A and pramipexole were generated using Charmm-GUI’s “Ligand Reader & Modeller” tool^66^. The loop grafting and optimization for modelling missing side chains and loops was performed in the ICM-Pro v3.9–2b molecular modeling and drug discovery suite (Molsoft LLC)^67^. The structurally conserved H29, A30 and Y31 at the N-terminus in the pramipexole bound D_3_R (PDB ID: 7CMU)^29^ were modeled using human FOB02–04A bound D_3_R as the template structure. The lobe in Gα_O_ protein was modeled using a human agonist-bound CB2-Gα_i_ structure (PDB ID: 6PT0)^68^. Structure regularization and torsion profile scanning were done using ICMFF force field^69^. The FOB02–04A-bound and pramipexole-bound structures of D_3_R complexes coupled to a Gα_O_βγ heterotrimer were then uploaded to the Charmm-GUI webserver^65,70^, where the starting membrane coordinates were determined by the PPM^70^ server using the Charmm-GUI interface. The complexes were then embedded in a lipid bilayer composed of 1,2-dipalmitoyl-sn-glycero-3-phosphatidylcholine (DPPC), 1,2-dioleoyl-sn-glycero-3-phosphatidylcholine (DOPC), and cholesterol (CHL1) following the recommended ratio of 0.55:0.15:0.30 respectively^71^. The FOB02–04A bound D_3_R coupled to a Gα_O_βγ heterotrimer contained 220 DPPC, 60 DOPC and 120 CHL1 lipids, 38818 water molecules, 112 sodium and 104 chloride ions. The pramipexole-bound D_3_R coupled to a Gα_O_βγ heterotrimer contained 220 DPPC, 60 DOPC and 120 CHL1 lipids, 37934 water molecules, 108 sodium and 102 chloride ions. Both systems were first subjected to 50000 steps of initial energy minimizations, then 60 ns of equilibration, followed by production runs of up to 500 ns. The simulations were carried out on GPU clusters at the University of Southern California’s High-Performance Computing Center. The temperature of 310 K and v-rescale thermostat algorithm were used during the production run^72^. The analyses of molecular dynamics trajectories was performed with MDTraj software package^73^.

#### Molecular Docking

The D_3_R structure was taken from the current work. The protein-stabilizing single-chain antibody scFv16 was removed from the D_3_R structure leaving the receptor protein subunit. The protein was processed via the addition and optimization of hydrogens and optimization of the side chain residues. Prior to conducting molecular docking, pramipexole underwent chiral definition and formal charge assignment. The compounds’ molecular models were created from their two-dimensional representations, and their three-dimensional geometry was refined using the MMFF-94 force field^74^. For docking simulations, a biased probability Monte Carlo (BPMC) optimization approach was employed, adjusting the internal coordinates of the compound based on pre-calculated grid energy potentials of the receptor^75^. The grid potentials, while preserving the receptor’s conformational state, considered receptor flexibility through the utilization of “soft” van der Waals potentials. All-atom docking was performed with the energy-minimized structure of FOB02–04A employing an effort value of 5. The ligand docking box was selected to encompass the extracellular half of the protein for potential grid docking. At least 10 independent docking runs with 3 conformations in each were conducted, starting from random conformations. Consistency among the docking results was determined by comparing ligand conformations from the best ten docking runs. The unbiased docking procedure did not rely on distance restraints or any predefined information regarding the ligand-receptor interactions. From these docking experiments, two top-scoring docking solutions, referred to as conformation A and conformation B, representing FOB02–04A bound to D_3_R complexes, were further refined. This refinement involved successive rounds of minimization and Monte Carlo sampling, focusing on the ligand conformation and including sidechain residues within 5 Å of the binding site. All the above-mentioned molecular modeling operations were performed in the ICM-Pro v3.9–2b molecular modeling and drug discovery suite (Molsoft LLC)^67^.
